# Criminal Mania

**Published:** 1896-04-25

**Authors:** F. M. F. Skene


### Criminal Mania.

By F. M. F. Skene.

### III.—THE DOCTOR'S DIFFICULTY.

We stated briefly in our last issue that the theory of criminal mania holds a very important place in that strong conviction of the maladministration of the law, and consequent injustice of its operations, which is widely spreading with most sinister effect among the so-called dangerous classes of the community. We have now to show that we have ample foundation for such a statement, as well as to illustrate by some typical instances the grave difficulties involved in it for members of the medical profession. The extent and vehemence of this underlying belief in the unequal and unjust exercise of the judicial power can only, perhaps, be rightly gauged by those who from circumstances—as in the case of the writer—have been brought into close contact with the "submerged" and with the inmates of prisons drafted from their midst, and to such observers it is clear that the sentiment of revolt among the people is most potent and universal, as regards the infliction of the capital penalty. Doubtless, they have their opinion also on the apparently capricious uncertainty which governs the distribution of lighter sentences, whether it be the peculiar anomalies of "Justices' justice" or the awards of higher courts, but these do not arouse in them the intolerable sense of wrong which springs from the unequal exercise of the power of life and death. They know that the scaffold is held by the law to be the inevitable doom of all persons clearly convicted of the crime of murder, yet the records of such cases, even in the recent past alone, show unmistakably that a considerable number of those whose guilt was no less distinctly proved than that of less fortunate criminals, did, in fact, escape the extreme sentence, while it was duly executed on others whose crimes had been committed under precisely similar conditions. It would not be difficult to make manifest the causes which lead to these inequalities in the administration of justice, but we are mainly concerned at present with the matter as it affects the responsibility of the medical experts, who are so often constituted virtual wielders of the power of life or death, and, in relation to them, some cases in point will be the most useful method of illustrating the subject.

Some years ago an elderly man in a good social position took measures to rid himself of his wife by one of the most deliberate, long-premeditated murders which was ever committed. He prepared a box as a receptacle for her mutilated remains, when they should have been reduced by his own hands to such fragments as could be placed within it, and he kept it by him, while he continued living quietly in the society of his victim, till a favourable opportunity occurred for her carefully-planned slaughter, when he fell upon her and slew her. All these facts were clearly proved, but it was very undesirable that a man in his peculiarly honoured position should be consigned to the gallows, and, although he was of necessity sentenced to death in the first instance, he was ultimately reprieved on the plea of insanity; he retired to a comfortable existence in a criminal lunatic asylum, where a letter from himself and other testimony made it perfectly plain that he was no more mad than we all may be considered to be who form part of the population—characterised by Carlyle as "mostly fools." About the same time when this murderer escaped his doom, a young man was executed under the following circumstances: He was a simple, hard-working labourer, passionately attached to an attractive but false-hearted girl who had formally engaged herself to marry him. A more eligible lover came in her way, and she speedily drove her betrothed into a condition of overmastering jealousy. The climax came when she walked into the room where he was sitting by the fire, and told him, with the most insulting scorn, that she loved his rival and meant to give herself to him, in spite of the vows she had exchanged with her legitimate lover. Stung to the quick, and literally maddened, in a moment of frenzy the young man took up the poker which lay close to his hand, and hit her with it on the back of her head; the blow did not kill her, and when her outraged assailant came to himself he repented with bitter remorse the brief fury to which he had given way when driven out of his senses by her cruel words. For a month the girl lived with a good prospect of complete recovery, but after that interval, sudden inflammation of the brain set in and she died, whereupon her unhappy lover was duly hanged. Can it be said that equal justice was meted out to these two men, or that the plea of temporary insanity would not have been far more justly employed in the case of the impulsive young man who had never harboured a murderous thought and simply succumbed to a moment's passion—than in that of the astute old miscreant who had deliberately planned and ingeniously carried out an assassination which he held to be necessary for his own comfort?

Perhaps an even more striking case was the following: A respectable tradesman who had borne a high character as a humane, kind-hearted man, was convicted of the murder of a woman he had married on very short acquaintance. He had killed her in a moment of blind rage, goaded to the deed after much long suffering by her infamous treatment of his children by his first wife, one of whom had just committed suicide, and by other sins against himself. The whole population of his native town and many influential persons, including the twelve jurymen who had recommended him to mercy, pleaded for his reprieve in vain. The judge, who had disregarded the plea for mercy which accompanied the verdict of the jury, was no doubt consulted by the Home Secretary, and the man's doom was sealed. At the very same time that he expiated his momentary frenzy on the scaffold, a man who had slain his wife, also after great provocation, was tried in another town by a different judge. On account of what in a French court would be called extenuating circumstances, this judge so arranged matters that he was able to sentence the criminal to one day's imprisonment only, with the result, as he had been some time in custody, that he walked out of the court then and there a free man. The crime of the two prisoners had been precisely the same, except that the provocation endured had been much greater in the case of the man who was executed than in that of the murderer who was discharged unpunished.

